# The Correlation Between Infant Head Shape in Craniometric Studies and Psychomotor Development Disorders

**DOI:** 10.3390/jcm14061985

**Published:** 2025-03-14

**Authors:** Natalia Zielińska, Maria Górska, Anna Skrzek, Agnieszka Dębiec-Bąk

**Affiliations:** 1 Faculty of Physiotherapy, Wroclaw University of Health and Sport Sciences, 35 I. Paderewskiego Avenue, 51-612 Wroclaw, Poland; natalia.zielinska@awf.wroc.pl (N.Z.); anna.skrzek@awf.wroc.pl (A.S.); 2 Children’s Rehabilitation Center Wroclaw, Bulwar Dedala 10a, 54-130 Wrocław, Poland; mariagorska131299@gmail.com

**Keywords:** infants, head shape, psychomotor development disorders, craniometric studies, plagiocephaly, brachycephaly

## Abstract

**Objectives**: The objective of this study was to analyze the correlation between muscle tone distribution disorders and asymmetry, with specific postnatal positional cranial deformities in infants. The study focused on assessing the level of unilateral occipital flattening and the extent of symmetrical occipital flattening. **Methods**: The study involved 60 infants aged between 1 and 5 months. Each infant was neurologically diagnosed and assigned to one of three groups: asymmetry, reduced muscle tone, or increased muscle tone. Each group consisted of 20 infants (10 girls and 10 boys). A MIMOS craniometer was used to measure the infants’ head shapes, calculating the cranial vault asymmetry (mm) and cranial index (%). The data were analyzed and processed using Statistica software and appropriate statistical tests. **Results**: The results revealed a correlation between asymmetry and reduced muscle tone, increased muscle tone, and specific positional head deformities in infants up to the age of 5 months.

## 1. Introduction

Motor development in an infant’s first year of life is typically described as a sequential progression of skills considered standard for developmental stages. These milestones are achieved in various postures, including lying, crawling, sitting, and standing. Abnormalities during fetal life or the perinatal period can result in motor development disorders and delays in reaching milestones, subsequently hindering the development of antigravity muscle function. The early diagnosis of such abnormalities is crucial for implementing appropriate interventions, which can mitigate or entirely prevent developmental delays and counteract the undesirable secondary consequences of motor disorders [1,2].

One developmental abnormality in infants is muscle tone disorder. Muscle tone is maintained through a feedback loop between the brain and the peripheral nervous system. Disruptions in this cycle can lead to atypical muscle tone, such as hypotonia or fluctuating tone [3].

Hypotonia, or reduced muscle tone, which is often observed proximally, disrupts the balanced distribution of tension. This leads to weakened trunk stabilization, preventing precise movements in distal body parts [3,4].

Hypertonia is defined as having an abnormally high muscle tone. Hypertonia that is evident from birth is relatively rare and often associated with severe neurological conditions, such as brain injury or metabolic disorders. Infants who are later diagnosed with spastic cerebral palsy often do not exhibit hypertonia during early life [3,4].

Another developmental abnormality is asymmetry, which is categorized as functional or structural. Functional asymmetry relates to emotions, perceptions, lateralization, and speech, while structural asymmetry involves the alignment of paired body parts, mass distribution, and organ positioning. Asymmetry is dynamic, with local asymmetry often transitioning into general asymmetry and structural asymmetry often transitioning into functional asymmetry, and vice versa. Asymmetrical head positioning frequently leads to asymmetry in other body parts [4,5].

The bones of the skull (neurocranium and viscerocranium) are connected by sutures that play a significant role during the following developmental stages: fetal, neonatal, infant, and early childhood. These sutures allow proper cranial growth and development over the first 6–8 years of life. Early-stage skull deformities primarily affect neurocranial structures. If there is no intervention to correct these deformities before the rapid development of facial structures then facial deformations may occur. The larger and more permanent the neurocranial deformation, the more pronounced the facial deformation becomes [6].

The most common cause of cranial deformities during growth is the influence of external modelling forces [7]. Positional cranial deformities are more prevalent than those caused by synostosis and are increasingly observed due to the preference for supine sleeping positions in infants. These deformities usually become apparent between the sixth and eighth weeks of life. Asymmetries less than one centimeter (measured diagonally) are classified as aesthetic. These become nearly invisible in later life, therefore, they do not require treatment during infancy. Asymmetries greater than two centimeters, however, are considered absolute indications for treatment [8].

Cranial deformities during development include (among others) positional plagiocephaly (unilateral occipital flattening) and positional brachycephaly (symmetrical occipital flattening).

**Figure 1:**
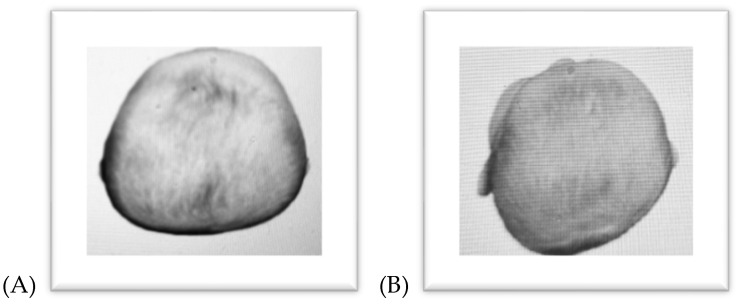
Positional head deformities brachycephaly (**A**), plagiocephaly (**B**), (own source).

Plagiocephaly, depending on its severity, manifests as unilateral occipital flattening and contralateral frontal flattening. Characteristic features include facial asymmetry, misalignment of the ears, and asymmetrical eye positioning (Figure 1). Factors contributing to plagiocephaly include multiple pregnancies, prematurity, male sex, and torticollis [5,9,10].

Brachycephaly is a cranial deformation characterized by a shortened anterior–posterior dimension and a widened biparietal diameter, without noticeable cranial asymmetry (Figure 1), [5,8,10].

One of the causes of brachycephaly is prolonged supine positioning during infancy [11]. The degree of brachycephaly is determined based on the cranial index. The normal values for this index typically range from 75% to 85% [12]. The values of the cranial index are reported differently in the literature; the main reason for these differences are, among other things, regional and country-specific variations. In the United States and Europe, a cranial index range of 72–81% is considered normal, while in Asia it is 80–93% [11].

## 2. Objective

The objective of this study was to analyze the correlation between muscle tone distribution disorders (reduced or increased muscle tone) and asymmetry, and specific postnatal positional cranial deformities in infants. The study focused on assessing the level of unilateral occipital flattening and the extent of symmetrical occipital flattening.

### Research Hypothesis

Asymmetry, reduced muscle tone, and increased muscle tone are associated with the head shape of infants up to the age of 5 months.

## 3. Study Materials

The study included 60 infants (30 girls and 30 boys) aged between 1 and 5 months (mean age: 3.03 months). The parents accompanied the infants during the examinations, and each parent confirmed that it was their infant’s first physiotherapy session and cranial measurement. Each infant had a prior diagnosis from a neurologist, classifying them into one of three research groups: asymmetry, reduced muscle tone, or increased muscle tone. Each group included 20 infants: 10 girls and 10 boys.

During the initial visit, each infant underwent measurement with a MIMOS company craniometer to determine their cranial vault asymmetry (CVA) and cranial index (CI). These assessments were conducted by physiotherapists at a Pediatric Physiotherapy Centre in Wroclaw and were systematically performed for all newly admitted patients.

All procedures performed in studies involving human participants are in accordance with the ethical standards of the institutional and/or national research committee, and with the 1964 Helsinki declaration and its later amendments, or with comparable ethical standards.

The inclusion criteria for this study included the following:

- A diagnosis by a neurologist (asymmetry, reduced muscle tone, or increased muscle tone), made no more than one week prior to the study.
- Aged between 1 and 5 months.
- No comorbid conditions.
- A birth term of between 39 and 40 weeks of gestation.
- An absence of cranial deformities caused by the birthing process.
- No prior physiotherapeutic interventions.
- Parental or legal guardian consent for participation in the study.

The exclusion criteria for this study included the following:

- A diagnosis of other conditions affecting the analyzed parameters.
- Previous physiotherapeutic interventions.
- A latex allergy.

## 4. Study Methods

### 4.1. Cranial Measurements

The primary tool used for the study was a MIMOS company craniometer. Each infant included in the study underwent two types of measurement: asymmetry and proportionality. All measurements were conducted in a physiotherapy clinic by the same examiner. During the procedure, one parent held the infant securely, facing the examiner.

As preparation for the measurement, an elastic band was placed around the infant’s head, ensuring the red arrow was aligned with the infant’s nasal line.

**Figure 2:**
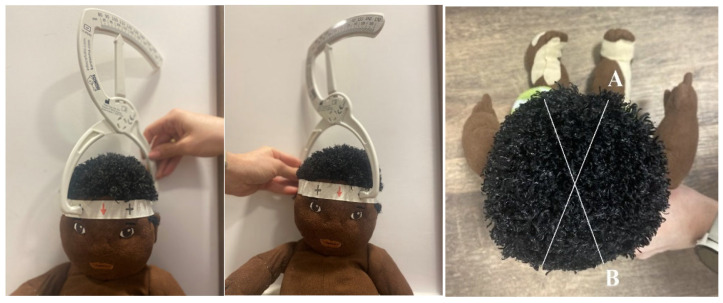
Method for measuring cranial asymmetry using a MIMOS company craniometer. The designated diagonals were the distances between the “+” mark on the right frontal bone and the “+” mark on the left occipital bone (measurement “A”) and the “+” mark on the left frontal bone and the “+” mark on the right occipital bone (measurement “B”).

The first part of the examination involved measuring the difference in length between two diagonal dimensions of the skull (labelled A and B) to determine cranial asymmetry (Figure 2). The measurements were then applied to the following formula:│A − B│ = Cranial Vault Asymmetry (CVA). The assessment of the level of cranial flattening was based on two factors: the CVA measurement and the infant’s age in months. The asymmetry measurement evaluated the level of positional deformation resulting from the unilateral flattening of the posterior part of the skull, known as plagiocephaly.

**Figure 3:**
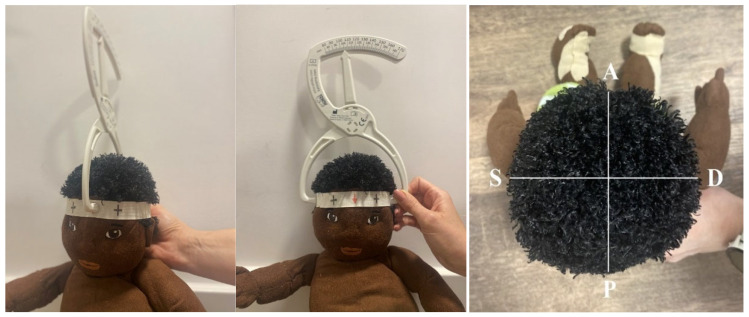
Proportion Measurement Schema. The schematic illustrates the two distances measured: (1) the widest point of the head, taken with the craniometer parallel to the face (SD measurement, sinistra/dextra) and (2) the longest point of the head, taken with the craniometer perpendicular to the face (AP measurement, anterior/posterior).

The second part of the study involved measuring cranial proportions. Two distances were measured: (1) SD (Latin: sinistra/dextra)—the widest point of the head—which was measured with the MIMOS company craniometer parallel to the face and (2) AP (Latin: anterior/posteriori)—the longest point of the head—which was measured with the cranial scanner perpendicular to the face (Figure 3). The following formula was used to calculate the cranial index (CI): $\frac{SD}{AP}*100$ = Cranial Index (%). The assessment of the flattening was based on two factors: the degree of flattening (CI) and the infant’s age in months. The proportion measurement evaluated the degree of positional deformation resulting from the central posterior flattening of the skull, known as brachycephaly.

### 4.2. Assessment of Psychomotor Development in Infants

The division into experimental subgroups was based on a neurologist’s diagnosis, which included an analysis of the quantity and quality of spontaneous and evoked motor activity. This study utilized assessments of muscle tone levels and body position symmetry at rest and during motor activities. Based on these evaluations, each child was assigned by the neurologist to one of three study groups: asymmetry, reduced muscle tone, or increased muscle tone.

### 4.3. Statistical Methods

The measurement results were subjected to a statistical analysis using STATISTICA PL version 13 software. The Shapiro–Wilk test was employed to confirm the normal distribution of the data. Basic descriptive statistics were calculated, including measures of the central tendency (mean, M) and standard deviation (SD). One-way ANOVA was used to compare the means across the three groups. The Pearson correlation analysis was performed to assess the correlations among the parameters studied. A significance level of *p* < 0.05 was adopted for all analyses.

## 5. Results

Table 1 summarizes the analyses of age, CVA, and CI for all infants, without division into subgroups.

Table 2 provides an analysis of the mean age, cranial vault asymmetry index (CVA), and cranial index (CI) values for each subgroup: asymmetry, reduced muscle tone, and increased muscle tone. A one-way ANOVA was used to compare the mean ages of these three subgroups. No statistically significant differences in age were observed among the subgroups (*p* = 0.86).

The asymmetry subgroup exhibited the highest mean CVA (15.4 ± 3.2). Both the reduced and increased muscle tone subgroups showed identical mean CVA values (4.5). The asymmetry subgroup differed significantly from the other two subgroups (*p* < 0.001), while no significant difference was found between the reduced and increased muscle tone subgroups (*p* = 1.000), (Table 3).

The asymmetry subgroup had the lowest mean CI (81.0 ± 6.3%). The highest mean CI was observed in the reduced muscle tone subgroup (93.7 ± 4.0%). Statistically significant differences were observed among all the subgroups for CI (Table 4).

By analyzing the correlations among the observed variables in the subgroups according to age, it was found that, in infants with asymmetry, the average CVA increased with age (Pearson’s r = 0.49; *p* = 0.029). This observation was statistically significant for this group (Table 5).

In the subgroup with reduced muscle tone, no statistically significant correlations were observed among age, CVA, and CI (Table 6).

In the subgroup with increased muscle tone, all observed variables were significantly correlated. CVA and CI exhibited an inverse correlation, indicating that as the CI increases, the average CVA decreases. Additionally, with the increase in age (months), a decline in the average CVA and a rise in the average CI were observed (Table 7).

The CVA increased with age in the asymmetry subgroup, but decreased in the reduced and increased muscle tone subgroups. Further correlation analyses indicated that the CI increased with age in the asymmetry subgroup, while it decreased in both the group with reduced muscle tone and the group with increased muscle tone.

In the asymmetry subgroup, both the CVA and CI indices increased. In the increased muscle tone subgroup, higher CVA correlated with lower CI, highlighting an inverse correlation.

A variance analysis considering gender revealed no statistically significant differences in the results. Gender does not significantly influence cranial asymmetry (plagiocephaly) or cranial proportions (brachycephaly).

## 6. Discussion

This study explored the correlation between psychomotor disorders in infants up to 5 months old and cranial shape anomalies. Correlations between diagnoses of asymmetry, reduced muscle tone, and increased muscle tone, and the type and extent of cranial deformities, such as plagiocephaly and brachycephaly, were investigated. The analyses underscored the value of early diagnosis; the early measurement of cranial deformation; and non-invasive interventions, such as physiotherapy and caregiving techniques.

The evaluation of the cranial deformation types and their severity revealed varied associations with diagnosed asymmetry and reduced or increased muscle tone. A statistically significant correlation was observed between plagiocephaly and age, as average CVA values increased with the infants’ age.

This correlation aligned with findings by Di Chiara et al. who demonstrated an age-related increase in unilateral occipital flattening due to reduced cranial molding potential. These findings underscore the importance of the early measurement and diagnosis of unilateral occipital flattening (plagiocephaly), which enhances the likelihood of achieving non-invasive reduction or the full correction of positional plagiocephaly, thereby preventing further asymmetry progression [13].

Bialocerowski et al. in a systematic review, highlighted that restricted cervical spine mobility may predispose infants to the development of plagiocephaly. By three months of age, most infants develop sufficient coordination and strength to hold their heads against gravity. Any internal or external factors limiting head movement during the first few months of life, including asymmetry, significantly increase the likelihood of cranial deformation in the form of plagiocephaly [14]. This heightened risk is particularly evident in preterm infants and those with impaired or delayed psychomotor development, as these factors delay independent head mobility [15]. Rogers et al. demonstrated that limited head mobility in early infancy, which is often caused by conditions such as torticollis, is a primary cause of plagiocephaly [16]. Similarly, Ohman et al. found that asymmetry and congenital torticollis may delay the achievement of early motor milestones, increasing the risk of cranial deformation [17]. Other researchers have also associated cervical spine mobility restrictions and torticollis with plagiocephaly [9,18,19].

Huang et al. in a study of 2118 infants under 4 months of age, emphasized the significant interplay between positional cranial deformations and motor abilities [7]. Sasaki et al. identified that head tilt due to asymmetry often accompanies mandibular deviation in the opposite direction, with mandibular deviation correlating to increased temporalis muscle volume on the same side. The facial asymmetry caused by unilateral positional occipital deformation may lead to changes in the development of the temporalis muscles [20].

An analysis of this study’s results indicated that the degree of unilateral occipital flattening (plagiocephaly) increased with age, suggesting that there are greater risks of structural impairments in affected children. Ghizoni et al. observed that, in children with plagiocephaly, frontal and occipital prominences develop on the side opposite the initial flattening. Ipsilateral ear and mastoid process displacement downward results in a parallelogram-shaped skull when viewed from above [21]. Kajdic et al. also noted mandibular deviation and bite changes in their study of infants [22].

This study found that bilateral occipital flattening (brachycephaly) correlates with both reduced and increased muscle tone. Kelly et al. showed that brachycephaly can cause several adverse effects, including angular changes in temporomandibular joint orientation, leading to malocclusion. They also found that brachycephaly may shift the head’s center of mass, disrupting the balance of cervical spine flexor and extensor muscles and impairing postural stability. Despite these findings, positional cranial deformations are often regarded as cosmetic issues [23].

Knight identified associations between brachycephaly and lower cognitive performance in adolescence, suggesting a link between positional cranial deformation and delayed cognitive and psychomotor development [24]. Bauriat et al. reached similar conclusions, explaining the delays through various mechanisms [6].

This study observed a CI increase with age and highlighted the serious consequences of untreated or late-diagnosed brachycephaly, as noted in other research. These findings underscore the importance of early diagnosis, early severity assessment, and the prompt implementation of conservative treatment to prevent further adverse changes.

This study also revealed a correlation between reduced muscle tone and central occipital flattening (brachycephaly). Infants with reduced muscle tone remain in the supine position longer due to reduced antigravity muscle capabilities, leading to multiple consequences, including brachycephaly, as confirmed by this study. Graham et al. similarly identified hypotonia as a risk factor for positional brachycephaly [25].

In this study, it was demonstrated that, as with reduced muscle tone, increased muscle tone also correlates with central occipital flattening (brachycephaly). Increased muscle tone generates structural and functional disruptions in the psychomotor development of infants. This was similarly observed by Kolehmainen et al. and Goo et al. who showed that abnormalities in muscle tone, including hypertonia, can affect daily activities, movement variability, joint mobility, growth, posture, and biomechanical alignment [3,26]. Choi et al. linked brachycephaly to malocclusion, sleep apnea, and postural abnormalities [27].

This study also demonstrated the efficacy of cranial measurements. Other researchers confirmed that objective anthropometric measurements are effective in assessing the severity of plagiocephaly [28,29]. Jung and Yun found that a diagonal length difference of 9–12 mm between the sides of the head indicates mild to moderate asymmetry, while differences exceeding 12 mm signify severe asymmetry [30].

Abboud et al. reported that satisfactory outcomes in deformational plagiocephaly can be achieved through conservative treatment when initiated early [31]. Unnithan and De Jesus emphasized that treating plagiocephaly primarily involves modifying the child’s positional behavior, although additional measures, such as helmet therapy, may be required in certain cases. The treatment of positional brachycephaly focuses on conservative methods, including physiotherapy, massage, and proper infant care [32].

Since Clarren et al. introduced helmet therapy, many researchers have confirmed its high efficacy as an adjunctive treatment for severe positional plagiocephaly [33]. Recommendations published in 2016 advocated the use of corrective helmets for severe positional deformities [23,34]. Di Chiara et al. demonstrated the effectiveness of external devices, such as cranial helmets (orthoses) and orthopedic bands, in correcting asymmetrical head shapes [13].

The increasing number of patients with positional cranial deformities underscores the importance of early diagnosis, parental education, and involvement in prevention and treatment processes. Preventive measures aim to reduce the time that infants spend in a supine position and provide an unrestricted environment to develop spontaneous and symmetrical motor functions emphasized that the early detection of plagiocephaly and early intervention was highly effective in correcting positional deformities [6,9].

The analyses conducted in this study, along with the findings of other authors, confirmed the relationship between positional head deformities and asymmetry, reduced muscle tone, and increased muscle tone. Although this study did not directly assess the impact of therapy on the improvement of muscle tone or cranial shape over time, considering these factors when planning the physiotherapy process may contribute to more effective and faster improvements in infants with such deformities.

## 7. Conclusions

1. Asymmetry and reduced or increased muscle tone are associated with cranial shape abnormalities in infants up to 5 months of age.
2. Reduced and increased muscle tone correlate most strongly with brachycephaly, with mean degrees of flattening increasing with age.
3. The sex of the infants is not a differentiating factor in the level of either brachycephaly or plagiocephaly.

## Figures and Tables

**Table 1:** Mean values of age, CVA, and CI across all infants.

Variable	N	Mean	SD	Var. Coeff. (%)
Age [months]	60	3.03	1.40	46.21
CVA [mm]	60	8.10	5.69	70.20
CI [%]	60	88.23	6.74	7.65

**Table 2:** Mean values of age, CVA, and CI in subgroups (based on neurological diagnoses).

Variable	Subgroup	N	Mean	SD
Age [months]	Asymmetry	20	2.90	1.45
CVA [mm]		20	15.40	3.19
CI [%]		20	81.00	5.07
Age [months]	Reduced muscle tone	20	3.05	1.39
CVA [mm]		20	4.45	1.76
CI [%]		20	93.70	4.01
Age [months]	Increased muscle tone	20	3.15	1.42
CVA [mm]		20	4.45	1.73
CI [%]		20	90.00	3.13

**Table 3:** Variation in the CVA: post-hoc probabilities (Bonferroni test).

Subgroup No.	Subgroup	1 15.40	2 4.45	3 4.45
1	Asymmetry		0.00	0.00
2	Reduced muscle tone	0.00		1.00
3	Increased muscle tone	0.00	1.00	

**Table 4:** Variation in the CI (%): post-hoc probabilities (Bonferroni test).

Subgroup No.	Subgroup	1 81.00	2 93.70	3 90.00
1	Asymmetry		0.000000	0.000000
2	Reduced muscle tone	0.000000		0.019703
3	Increased muscle tone	0.000000	0.019703	

**Table 5:** Analysis of the correlations among age, CVA, and CI in the asymmetry subgroup.

Variable	Subgroup = Asymmetry		
	The Correlation Coefficients Are Significant at *p* < 0.05		
	Age	CVA	CI
Age	1.000	0.4886	0.2009
		*p* = 0.029	*p* = 0.396
CVA	0.4886	1.0000	0.4303
	*p* = 0.029		*p* = 0.058
CI	0.2009	0.4303	1.0000
	*p* = 0.396	*p* = 0.058	

**Table 6:** Analysis of the correlations among age, CVA, and CI in the reduced muscle tone subgroup.

Variable	Subgroup = Reduced Muscle Tone The Correlation Coefficients Are Significant at *p* < 0.05		
	Age	CVA	CI
Age	1.0000	−0.2239	0.3507
		*p* = 0.343	*p* = 0.130
CVA	−0.2239	1.0000	−0.0097
	*p* = 0.343		*p* = 0.968
CI	0.3507	−0.0097	1.0000
	*p* = 0.130	*p* = 0.968	

**Table 7:** Analysis of the correlations among age, CVA, and CI in the increased muscle tone subgroup.

Variable	Subgroup = Increased Muscle Tone The Correlation Coefficients Are Significant at *p* < 0.05.		
	Age	CVA	CI
Age	1.0000	−0.5624	0.6613
		*p* = 0.010	*p* = 0.001
CVA	−0.5624	1.0000	−0.5441
	*p* = 0.010		*p* = 0.013
CI	0.6613	−0.5441	1.0000
	*p* = 0.001	*p* = 0.013	
